# MicroRNA-128-3p regulates mitomycin C-induced DNA damage response in lung cancer cells through repressing *SPTAN1*

**DOI:** 10.18632/oncotarget.12300

**Published:** 2016-09-28

**Authors:** Rui Zhang, Chang Liu, Yahan Niu, Ying Jing, Haiyang Zhang, Jin Wang, Jie Yang, Ke Zen, Junfeng Zhang, Chen-Yu Zhang, Donghai Li

**Affiliations:** ^1^ State Key Laboratory of Pharmaceutical Biotechnology, Nanjing Advanced Institute for Life Sciences(NAILS), School of Life Sciences, Nanjing University, Nanjing, Jiangsu 210093, China; ^2^ Jiangsu Engineering Research Center for microRNA Biology and Biotechnology, School of Life Sciences, Nanjing University, Nanjing, Jiangsu 210093, China

**Keywords:** lung cancer, MicroRNA, mitomycin C, spectrin, DNA repair

## Abstract

The DNA damage response is critical for maintaining genome integrity and preventing damage to DNA due to endogenous and exogenous insults. Mitomycin C (MMC), a potent DNA cross-linker, is used as a chemotherapeutic agent because it causes DNA inter-strand cross-links (DNA ICLs) in cancer cells. While many microRNAs, which may serve as oncogenes or tumor suppressors, are grossly dysregulated in human cancers, little is known about their roles in MMC-treated lung cancer. Here, we report that miR-128-3p can attenuate repair of DNA ICLs by targeting *SPTAN1* (αII Sp), resulting in cell cycle arrest and promoting chromosomal aberrations in lung cancer cells treated with MMC. Using computational prediction and experimental validation, *SPTAN1* was found to be a conserved target of miR-128-3p. We then found that miR-128-3p caused translational inhibition of *SPTAN1*, reducing its protein level. *SPTAN1* repression via miR-128-3p also induced cell cycle arrest and chromosomal instability. Additionally, miR-128-3p significantly influenced interaction of the αII Sp/FANCA/XPF complex, thus limiting DNA repair. In summary, the results demonstrate that miR-128-3p accelerates cell cycle arrest and chromosomal instability in MMC-treated lung cancer cells by suppressing *SPTAN1*, and these findings could be applied for adjuvant chemotherapy of lung cancer.

## INTRODUCTION

Mitomycin C (MMC) is an anti-tumor drug widely used in clinical cancer chemotherapy [1]. Use of MMC has been associated with a response rate of 20% in patients with non-small cell lung cancer (NSCLC), which is the most common malignant disease worldwide. Combination of MMC with ifosfamide in a previous phase 2 study of NSCLC achieved an overall response rate to chemotherapy of 43% [2]. Monofunctional and bifunctional alkylation of DNA by MMC has been proven to produce a single covalent DNA adduct and DNA inter-strand cross-links (DNA ICLs) [3], and these DNA pathological changes constitute the molecular basis of the cytotoxicity of MMC toward tumor cells. A later study reported that its cytotoxicities and derivatives are closely related to the observed high frequencies of DNA ICLs [4]. However, the molecular mechanism by which MMC, as one of 3 most active agents, functions with microRNAs (miRNAs) to treat NSCLC patients remains unclear.

MiRNAs are a class of endogenous non-coding RNAs, typically 22 nucleotides in length, that function primarily by targeting the 3′-untranslated region (3′-UTR) of specific mRNAs and hence silence gene expression through either translational repression or direct mRNA degradation [5]. Such posttranscriptional gene regulation mechanisms allow miRNAs to control a wide range of biological processes, including cell proliferation and differentiation, migration, apoptosis, development and metabolism [6]. Accumulating evidence has shown aberrant miR-128 expression in tissues and blood from patients with many types of malignant tumors [7–12]. Volinia et al. examined 540 different types of malignant tumor samples by miRnome analysis and found significant increases in expression of miR-128 in colon, lung and pancreatic cancer [7]. In another report, *in situ* hybridization revealed that miR-128 expression is decreased in chemoresistant tumor tissues but increased in chemosensitive tissues, and the level of miR-128 expression in breast cancer tissues was correlated with patient response to novel adjuvant chemotherapy and survival [13].

Spectrin is a multifunctional protein. In addition to its primary role in maintaining the mechanical properties of cell membranes, it has been reported to be involved in many biological pathways such as the cell cycle, DNA repair, cell adhesion and spreading [14, 15]. Previous studies have demonstrated that nonerythroid αII spectrin (αII Sp) is present in the mammalian cell nucleus, where it plays an important role in repair of DNA ICLs and is critical for chromosome stability [16–19]. αII Sp functions together with the DNA ICL repair proteins XPF and FANCA to localize at nuclear foci after DNA ICL damage [16, 18, 20]. A recent report has shown β2 spectrin deficiency disturbs chromosome stability [21], and a number of studies have shown that spectrin is an essential regulator in a variety of cancers [22–26].

In the present study, we found that *SPTAN1* is predicted to be a direct target of miR-128-3p. An inverse correlation between miR-128-3p expression and αII Sp protein level in lung cancer treated with MMC was confirmed experimentally. MiR-128-3p was found to disrupt the cell cycle in lung cancer by targeting *SPTAN1*. Furthermore, the decreased level of αII Sp protein resulted in marked chromosomal instability and limited its function of recruiting XPF and FANCA for DNA repair.

## RESULTS

### MMC-induced chromosomal aberrations and miR-128-3p expression in lung cancer cells

**Figure 1:**
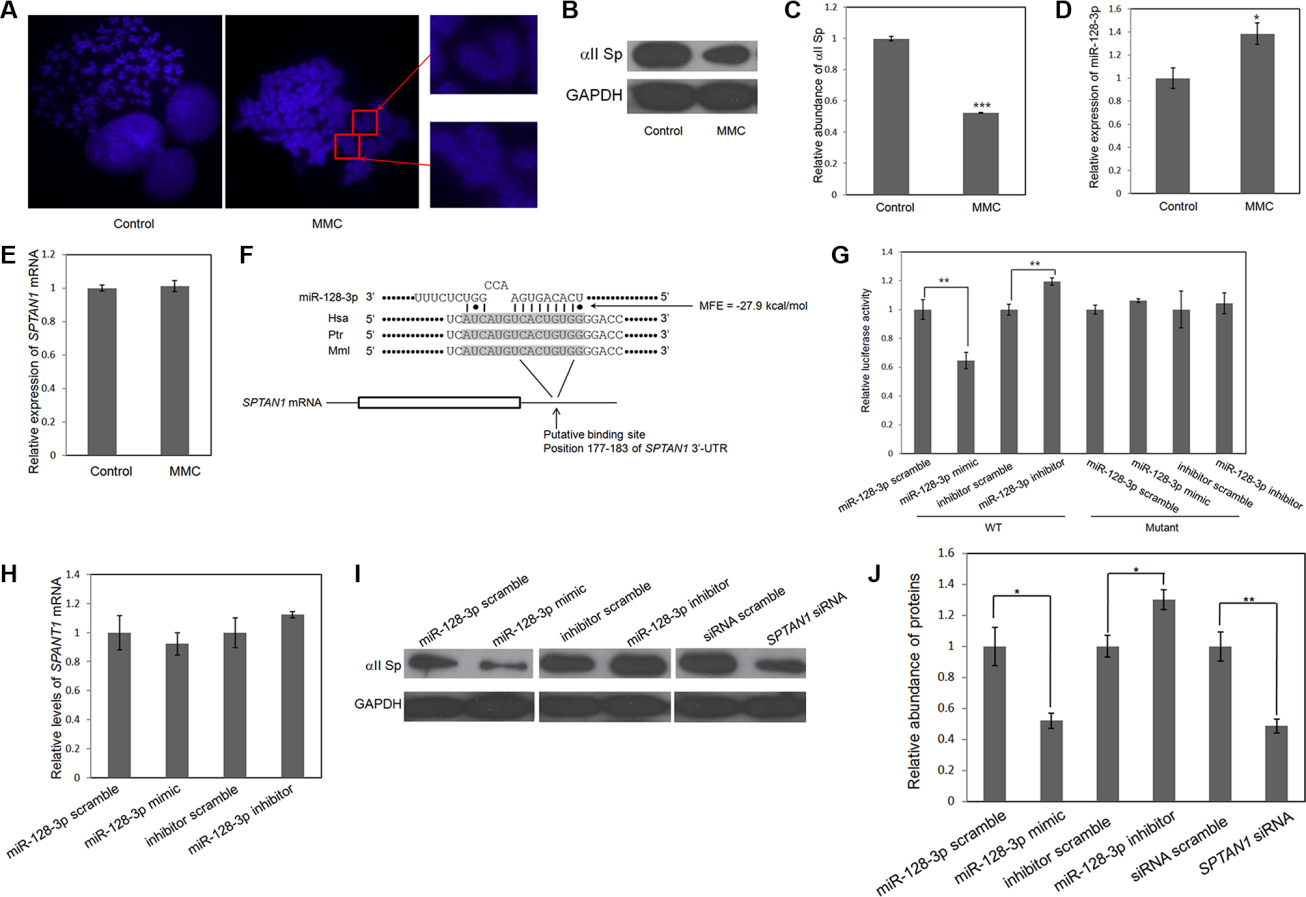
MiR-128-3p directly targets *SPTAN1* via translational repression (**A**) MMC treatment leads to an increase in chromosomal aberrations. (**B**, **D** and **E**) The effect of MMC on protein and mRNA levels of *SPTAN1* and miR-128-3p expression. (**C**) Relative abundance of the αII Sp protein level in (B). (**F**) Schematic description of miR-128-3p binding site in the *SPTAN1* 3′-UTR. Paired bases are indicated by a black short line, and G:U pairs are indicated by dots. The predicted free energy of each hybrid is indicated. Sequence alignment of putative miR-128-3p binding sites across species are marked by a gray background. (**G**) A firefly luciferase reporter containing either wild-type (WT) or mutant *SPTAN1* 3′-UTR was co-transfected into A549 cells with scrambled ncRNA or miR-128-3p mimic or inhibitor. For comparison, luciferase activity in ncRNA-transfected cells was set at 1. The y-axis shows arbitrary units representing the relative luciferase activity. (**H**) Quantitative RT-PCR analysis of *SPTAN1* mRNA levels in A549 cells transfected with scrambled ncRNA, miR-128-3p mimic or miR-128-3p inhibitor. (**I**) Western blot analysis of αII Sp protein levels in A549 cells transfected with scrambled ncRNAs, miR-128-3p mimic, miR-128-3p inhibitor or *SPTAN1* siRNA. (**J**) Relative abundance of αII Sp in (I). The results are presented as the mean ± SE of three independent experiments. *p < 0.05, ^*^p < 0.01, ^**^*p < 0.001.

In agreement with previous studies, MMC significantly induced chromosomal aberrations in lung cancer cells compared to a control group without MMC treatment (Figure 1A). To investigate the molecular mechanism by which MMC alters chromosomal stability to kill cancer cells, the levels of miR-128-3p and *SPTAN1* expression were detected, as abundant data indicate their important functions in chromosomal instability, DNA ICLs and cancer [12, 16, 27, 28]. As shown in Figure 1B, 1C and 1E, the protein level of αII Sp decreased by 47%, whereas that of miR-128-3p increased by ~1.4-fold compared to the corresponding control. These results indicate that miR-128-3p and αII Sp are reversely correlated. Importantly, mRNA expression of *SPTAN1* was unchanged (Figure 1D).

### MiR-128-3p targets *SPTAN1* via translational repression

MiRNAs are crucial regulators in lung cancer. Multiple target prediction programs were applied for determining the potential targets of miR-128-3p. Based on the species conservation and minimum free energy (MFE) of their binding sites as well as their cancer/DNA damage response correlations, *SPTAN1* was highlighted for further investigation. Figure 1F illustrates the predicted interaction of miR-128-3p and the target site in the *SPTAN1* 3′-UTR (MFE = −27.9 kcal/mol). A luciferase assay was performed to examine whether *SPTAN1* is a direct target of miR-128-3p. The entire 3′-UTR of *SPTAN1* placed in a reporter plasmid downstream of firefly luciferase. The resulting plasmid was transfected into A549 cells along with a transfection control plasmid and a miR-128-3p mimic or scrambled ncRNA. As hypothesized, compared to treatment with scrambled ncRNA, the miR-128-3p mimic decreased the luciferase activity to 35% of that of the reporter containing the miR-128-3p binding site, whereas a miR-128-3p inhibitor increased activity by 19%. We generated mutations in the corresponding complementary seed sites in the 3′-UTR of *SPTAN1* to eliminate the predicted miR-128-3p binding. Mutations in complementary seed sites almost fully rescued the repression of reporter activity caused by the miR-128-3p mimic (Figure 1G). Collectively, these findings strongly indicate that miR-128-3p can directly recognize the binding site in the 3′-UTR of *SPTAN1* and mediate posttranscriptional inhibition of the gene.

Theoretically, miRNAs silence gene expression by either translational repression or direct mRNA degradation. Thus, we next sought to confirm which mechanism miR-128-3p uses to modulate *SPTAN1* expression. We transfected A549 cells with equal doses of scrambled ncRNA, miR-128-3p mimic or miR-128-3p inhibitor and analyzed *SPTAN1* mRNA expression by RT-PCR at 24 h post-transfection. *SPTAN1* mRNA expression in all miR-128-3p mimic/inhibitor-transfected cells remained unchanged compared to that in all corresponding ncRNA-transfected cells (Figure 1H). However, we repeated the above experiments and determined whether overexpression or knockdown of miR-128-3p had an impact on the level of αII Sp protein by western blotting at 24 h post-transfection. Cells transfected with the miR-128-3p mimic showed a level of αII Sp protein that was reduced to almost half of that of cells transfected with scrambled ncRNA; in contrast, the protein level of αII Sp increased by 30% in miR-128-3p inhibitor-transfected cells compared to scrambled ncRNA-transfected cells (Figure 1I and 1J). We also transfected A549 cells with *SPTAN1* siRNA and siRNA scramble, which showed a 51% decrease and the same effect as observed in the miR-128-3p mimic-transfected cells (Figure 1I and 1J). Similar results were obtained in H1975 lung cancer cells (Supplementary Figure S1). These findings demonstrate that miR-128-3p is capable of regulating *SPTAN1* through translational repression.

### Up-regulation of miR-128-3p causes G2/M arrest and chromosomal instability

**Figure 2:**
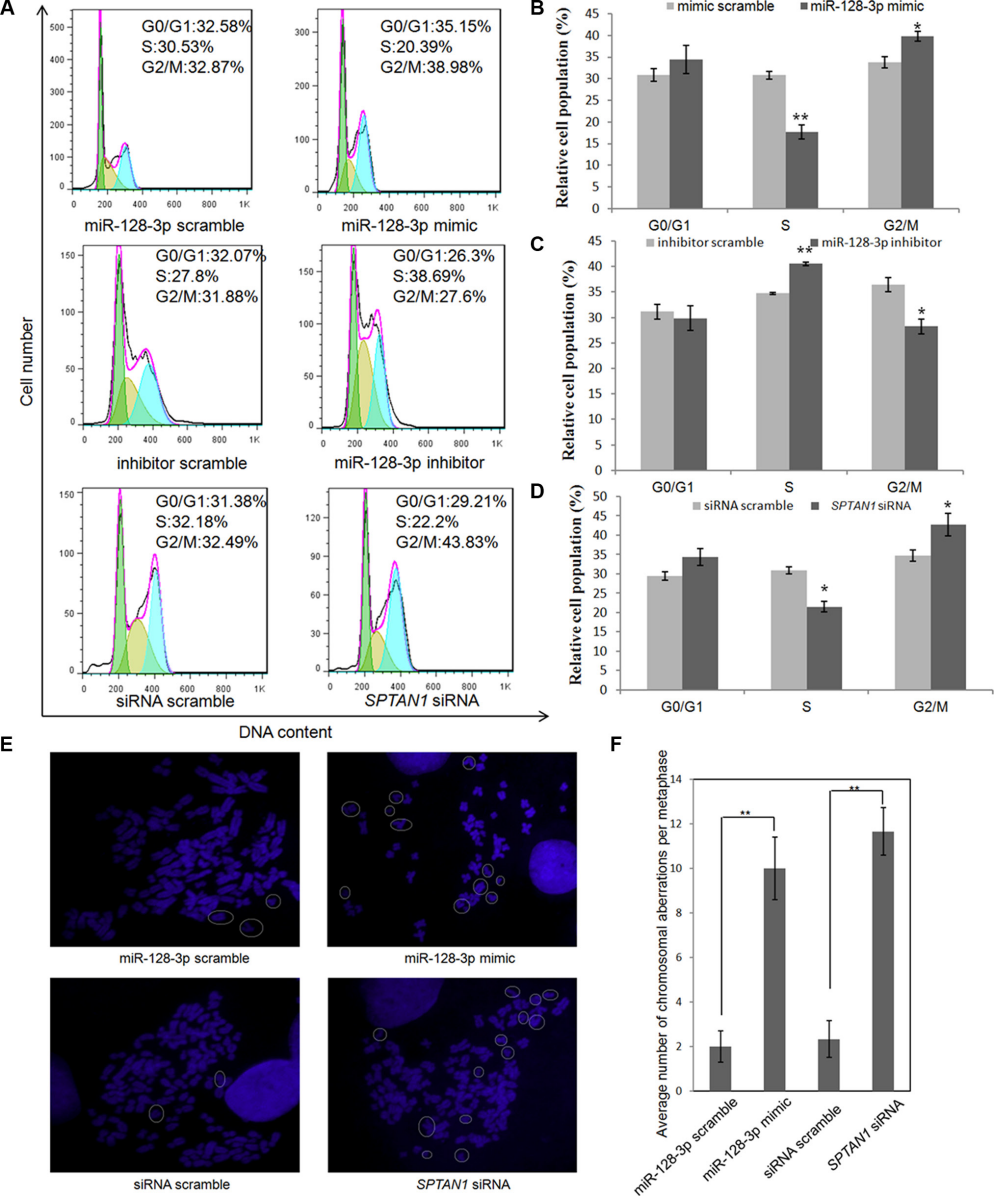
MiR-128-3p regulates the cell cycle and chromosomal aberrations (**A**) A549 cells were transfected with synthetic RNAs and subsequently treated with MMC; the percentages of cells in G0/G1, S and G2/M phases were determined by flow cytometry. (**B**, **C** and **D**) Bar graphs illustrate quantification of the cell cycle. (**E**) The effect of scrambled ncRNAs, miR-128-3p mimic and *SPTAN1* siRNA on chromosomes in A549 cells. Circles indicate chromosomal aberrations. (**F**) One hundred metaphase spreads were scored for chromosomal aberrations, and the average number of chromosomal aberrations per metaphase from three independent experiments is shown. The results are presented as the mean ± SE of three independent experiments. *p < 0.05, ^*^p < 0.01.

To investigate whether miR-128-3p and *SPTAN1* can affect the cell cycle after MMC-mediated DNA ICL damage, miR-128-3p was overexpressed or knocked down in A549 cells, and the cells were examined by flow cytometry (Figure 2A). Gain of function of miR-128-3p following mimic transfection resulted in an increase in the population of cells in G2/M phase, with a concomitant decrease in the fraction of cells in S phase (Figure 2B). Figure 2A shows a representative experiment in which 32.87% of miR-128-3p scramble-treated cells were in G2/M phase but 38.98% of miR-128-3p mimic-treated cells were in G2/M phase. Transfection with siRNA against αII Sp yielded an effect similar to that obtained via overexpression of miR-128-3p (Figure 2D). In the siRNA experiment, the G2/M-phase fractions of the siRNA scramble- and siRNA-treated groups were 32.49% and 43.83%, respectively (Figure 2A and 2D). In contrast, knockdown of miR-128-3p in A549 cells by a miR-128-3p inhibitor led to a reduction in cells in G2/M phase (Figure 2C). The percentage of inhibitor scramble-treated cells in G2/M phase was 31.88%, and that of miR-128-3p inhibitor-transfected cells was 27.6% (Figure 2A). The S-phase fractions of the control and miR-128-3p inhibitor groups were 27.8% and 38.69%, respectively. These results indicate that miR-128-3p blocked cell cycle progression by arresting cells in G2/M phase. Apoptosis analysis of lung cancer cells showed 2.8–8.1% apoptotic cells resulting from the same treatment.

To further identify the influence of miR-128-3p on chromosomal stability, overexpression or knockdown of miR-128-3p in A549 cells was evaluated for effects on chromosomal morphological changes using metaphase spread analysis. Metaphase spreads of A549 cells transfected with miR-128-3p mimic showed an obvious increase in chromosomal aberrations compared to ncRNA-transfected cells. *SPTAN1* siRNA showed a similar effect as the miR-128-3p mimic. These results suggest that miR-128-3p promoted chromosome instability by silencing *SPTAN1* (Figure 2E and 2F).

### MiR-128-3p destroys αII Sp/FANCA/XPF interaction by reducing αII Sp levels

**Figure 3:**
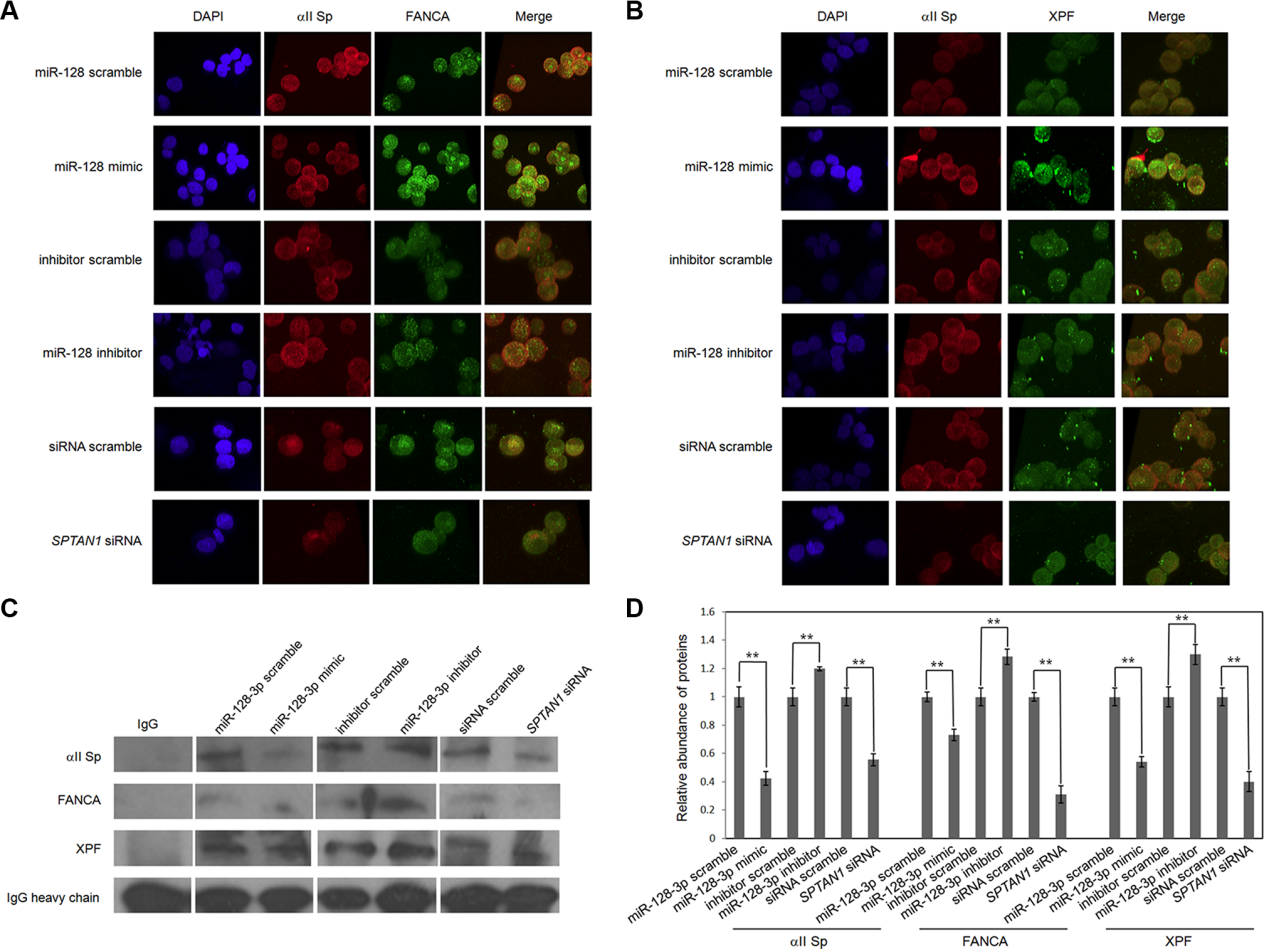
MiR-128-3p disrupts co-localization and interaction of FANCA, XPF and αII Sp (**A**) Co-localization of FANCA and αII Sp. Cells were stained with anti-αII Sp (red) and anti-FANCA (green) antibodies, and nuclear DNA was counterstained with DAPI (blue). (**B**) Co-localization of XPF and αII Sp. Cells were stained with anti-αII Sp (red) and anti-XPF (green) antibodies, and nuclear DNA was counterstained with DAPI (blue). (**C**) Interaction of αII Sp, XPF and FANCA. Co-IP was carried out using an anti-αII Sp antibody or IgG (as a control), and western blot analyses were performed using an anti-αII Sp, anti-FANCA or XPF antibody. (**D**) Bar graphs show the relative abundances of αII Sp, FANCA or XPF. The results are presented as the mean ± SE of three independent experiments. ^*^p < 0.01.

αII Sp is a crucial recruiter of FANCA and XPF in repair of DNA ICLs. To determine whether miR-128-3p influences interaction of the αII Sp/FANCA/XPF complex by inhibiting *SPTAN1*, immunofluorescent co-localization and co-immunoprecipitation (co-IP) experiments were performed. Figure 3A and 3B show an interruption of co-localization of FANCA or XPF with αII Sp in miR-128-3p mimic-transfected cells compared to ncRNA-transfected cells. Conversely, co-localization of FANCA or XPF with αII Sp in miR-128-3p inhibitor-transfected cells was greatly enhanced compared to that in inhibitor scramble-transfected cells. Knockdown of αII Sp by siRNA also led to loss of co-localization of FANCA or XPF with αII Sp (Figure 3A and 3B). Interestingly, FANCA protein levels in HeLa cells were reportedly stable in another study [17].

Co-IP were undertaken to ascertain whether miR-128-3p regulate the interaction between αII Sp and FANCA or XPF. As Figure 3C and 3D showed, cells transfected with miR-128-3p mimic made 58%, 27% or 46% reduction in protein levels of αII Sp, FANCA or XPF respectively relative to the cells transfected with ncRNAs. Apparently, miR-128-3p inhibitor boosted the abundances of αII Sp, FANCA or XPF (20%, 28% or 30%). *SPTAN1* siRNA and miR-128-3p mimic have similar effects to their interaction of the complex. Thus, these results indicate miR-128-3p breaks the formation of αII Sp/FANCA/XPF complex by eliminating αII Sp.

## DISCUSSION

MMC exhibits activity against NSCLC, a mechanism that involves cross-linking of inter-strand DNA to prevent DNA replication and inhibit tumor cell division. As a structural protein, αII Sp is implicated in the repair of DNA ICLs. However, the relationship between miRNAs and *SPTAN1* in DNA repair and the potential function by which MMC inhibits cancer cells is poorly understood. In this study, we describe a novel function for miR-128-3p, whereby regulation of chromosomal stability and cell cycle progression occur through *SPTAN1* in lung cancer cells treated with MMC. This conclusion is supported by several lines evidence: increased expression of miR-128-3p and decreased expression of *SPTAN1* in lung cancer cells treated with MMC; a putative *SPTAN1* binding site in the 3′-UTR that is subject to miR-128-3p regulation; overexpression of miR-128-3p augmented MMC-mediated chromosomal instability and cell cycle arrest of G2/M phase in lung cancer cells; these biological dysfunctions were caused by disruption of the αII Sp/FANCA/XPF complex induced by miR-128-3p. Completely different from the traditional function of DNA ICLs caused by MMC, the miR-128-3p-*SPTAN1* axis is a novel molecular mechanism for inhibiting the repair of DNA ICLs and could serve as an excellent potential auxiliary to treat lung cancer.

It has been found that aberrant expression of miR-128 occurs in malignant tumors, and this miRNA has been identified as a key regulator of oncogenic properties. Reduced miR-128 was first found in glioblastoma [29], and miR-128 is involved in multiple signal pathways associated with head and neck squamous cell carcinoma progression and growth [30]. MiR-128 is also down-regulated in glioma tissue and serum and could be used as potential biomarker in glioma identification, early diagnosis, classification and prognosis prediction [31]. MiR-128 targets ZEB1 in prostate cancer, and the miR-128-*ZEB1* axis could be a promising prognostic and therapeutic target for future prostate cancer therapy. MiR-128 promotes proliferation in osteosarcoma cells by down-regulating *PTEN* [32]. Abnormal expression of miR-128 contributes to the malignant phenotypes of cancer cells, such as proliferation [33], cell motility, invasion [34, 35], apoptosis [36] and self-renewal [37]. Here, we note that overexpression of miR-128 remarkably hindered DNA repair to contribute to the anti-cancer function of MMC and sensitized MMC-mediated chemotherapy in lung cancer.

It is well believed that MMC is an important effector of cell cycle arrest and chromosomal stability. For example, the percentage of cells containing chromosomal aberrations was significantly higher among PHF9-knock-down cells than among control cells in the presence of MMC [38]. Previous studies have shown that MMC causes a significant increase in the number of chromosomal aberrations, which could be related to a defect in the repair of DNA ICLs [27]. It has been reported that Bid-deficient cells have a defect in S or G2/M phase cell-cycle arrest upon treatment with replicative stress-inducing drugs such as MMC [39]. MMC also induces cell cycle arrest at clinically relevant doses (1 μg/ml) [40]. In addition to the above-mentioned findings, we here present the first evidence that miR-128-3p enhances MMC-induced chromosomal aberrations and cell cycle arrest at G2/M phase by targeting *SPTAN1*. Both miR-128-3p and αII Sp are essential for the function of MMC in causing chromosomal aberrations and cell cycle arrest.

Fanconi Anaemia (FA) is an inherited recessive disease caused by mutations in at least 15 different genes. Cellular hypersensitivity to cross-linking agents such as MMC is observed in FA, a trait that has been used as a diagnostic marker for FA patients. There is also biochemical evidence showing that FANCA, FANCC and FANCG bind *in vitro* to psoralen cross-linked DNA in a complex with human αII Sp [18]. Moreover, αII Sp is present in the mammalian cell nucleus, where it has an important function in DNA ICL repair and chromosome stability. αII Sp has been shown to recruit DNA repair-associated proteins (FANCA and XPF) to nuclear foci induced by DNA ICLs and to be crucial for chromosome stability [18, 20, 27]. Formation of the αII Sp/FANCA/XPF complex is involved in initial damage recognition and the incision steps of the repair process [20, 41, 42]. To date, however, there is no evidence that miRNAs participate in αII Sp regulation or adjust its function in lung cancer chemotherapy. Here, we demonstrate that overexpression of miR-128-3p down-regulates *SPTAN1* expression via translational repression and results in the failure to recruit FANCA and XPF to facilitate chromosomal instability after MMC-induced DNA ICL damage in lung cancer cells. MMC treatment not only cross-links DNA but also inhibits expression of *SPTAN1* through miR-128-3p to disrupt recruitment of DNA repair-associated proteins (FANCA and XPF), with dual destructive effects in lung cancer cells. This is a novel pathway for interrupting repair of DNA ICLs to enhance the anti-cancer function of MMC.

Although FA proteins can stabilize αII Sp, in FA cells, which have significant αII Sp deficiency, αII Sp can be cleaved into small segments by μ-calpain, but αII Sp is not affected by alternative RNA splicing [19, 43]. However, in both normal and cancer cells, changes in the level of αII Sp protein induced by MMC should be caused by a novel miRNA-related pathway different from the above-mentioned μ-calpain degradation system. Additionally, the anti-αII Sp antibody used in this study detected αII Sp in the cytoplasm and nucleus; thus, in-depth analysis is required in the near future. In addition, it remains unclear whether the these *SPTAN1* regulation pathways cooperate together to function under certain conditions. MiRNAs have potential pleiotropic effects, and many miRNAs can influence a single target gene. Therefore, other miRNAs or other miR-128-3p target genes might be involved in this process.

Taken together, we extend the current knowledge by highlighting the role of miR-128-3p in the sensitivity of lung cancer to chemotherapy. The miR-128-3p-*SPTAN1* axis provides a novel avenue for understanding the mechanism of chemosensitivity, and miR-128-3p could be a candidate molecular target for improving the efficacy of lung cancer chemotherapy.

## MATERIALS AND METHODS

### Cell culture and treatment of cells with MMC

A549 human lung adenocarcinoma cell line was purchased from the Shanghai Institute of Cell Biology, Chinese Academy of Sciences (Shanghai, China). The A549 cells were cultured in DMEM supplemented with 10% fetal bovine serum (FBS) (GIBCO). All cells were incubated in a 5% CO_2_, 37^°^C, water-saturated atmosphere. Synthetic RNAs-transfected cells were treated with 400 nM MMC (Sigma-Aldrich, USA) 24 h after transfection, as previously described [27].

### Overexpression or knockdown of miR-128-3p

Synthetic miR-128-3p mimic, miR-128-3p inhibitor and scrambled negative control RNAs (mimic scramble and inhibitor scramble) were purchased from GenePharma (Shanghai, China). Cells were seeded in 6-well plates and were transfected with the following day using Lipofectamine 2000 (Invitrogen, Carlsbad, CA, USA), according to the manufacturer's instructions. For each well, equal doses of miR-128-3p mimic, miR-128-3p inhibitor or scrambled ncRNA were added. Cells were collected 24 h after transfection for further analysis.

### RNA isolation and quantitative RT-PCR

Total RNA was extracted from the cultured cells using TRIzol Reagent (Invitrogen, Carlsbad, CA, USA) according to the manufacturer's instructions. For quantitative RT-PCR analysis of *SPTAN1* and GAPDH, 1 μg of total RNA was reverse transcribed to cDNA with oligo (dT) and Thermoscript (TaKaRa). Real-time PCR for *SPANT1* and GAPDH was performed on an Applied Biosystems 7300 Sequence Detection System (Applied Biosystems) using SYBR green dye (Invitrogen, Carlsbad, CA, USA). The sequences of the sense and antisense primers used for amplification of *SPTAN1* and GAPDH were as follows: *SPTAN1* (sense): 5′-TACGAGAATGTGAGGACGTGA-3′, *SPTAN1* (antisense): 5′-CATGAGCAGCCATATCTGTTTGA-3′; GAPDH (sense): 5′-GATATTGTTGCCATCAATGAC-3′, GAPDH (antisense): 5′-TTGATTTTGGAGGGATCT CG-3′.

Assays to quantify mature miR-128-3p were carried out using TaqMan miRNA probes (Applied Biosystems). Briefly, 1 μg of total RNA was reverse-transcribed to cDNA using AMV reverse transcriptase (TaKaRa) and a stem-loop RT primer (Applied Biosystems). Real-time PCR was performed using a TaqMan PCR kit on an Applied Biosystems 7300 Sequence Detection System (Applied Biosystems). All reactions, including no-template controls, were run in triplicate. After the reactions, the CT values were determined using fixed threshold settings. In the experiments presented here, miRNA expression in cells was normalized to U6. The relative amount of miR-128-3p to internal control U6 was calculated with the equation 2^−ΔC^_T_, in which ΔC_T_ = C_T__miRNA_ − C_T__U6_.

### Target predictions

Target genes were predicted with three algorithms from TargetScan (http://genes.mit.edu/targetscan/), miRWalk (http://www.umm.uni-heidelberg.de/apps/zmf/mirwalk/index.html) and miRanda (http://www.microrna.org).

### Plasmid construction and luciferase assay

The entire 3′-UTR or mutant of *SPANT1* (Invitrogen, Carlsbad, CA, USA) were inserted into the p-MIR-report plasmid (Ambion). For luciferase reporter assays, A549 cells were cultured in 24-well plates, and each well was transfected with 1 μg of firefly luciferase reporter plasmid, 1 μg of β-galactosidase expression vector (Ambion), and equal amounts of scrambled ncRNA, miR-128-3p mimic, or miR-128-3p inhibitor using Lipofectamine 2000 (Invitrogen, Carlsbad, CA, USA). The β-galactosidase vector was used as a transfection control. At 24 h post-transfection, cells were assayed using luciferase assay kits (Promega). Data depicted are representative of three independent experiments.

### Protein extraction and western blotting

Whole cell extracts was obtained at 24 h post-transfection. All protein level was quantified by western blot analysis of whole cell extracts using anti-αII Sp antibody (Santa Cruz Biotechnology). These samples were normalized by probing the same blots with a GAPDH antibody.

### Cell cycle analysis

Cells were harvested and washed twice in PBS at room temperature and resuspended at 2 × 10^6^ cells/ml in PBS. For propidium iodide staining, washed cells were fixed in 70% ethanol at −20°C overnight and the rest of the steps were performed according to the manufacture's procedure (Molecular Probes Inc.). Cells were washed twice in PBS, resuspended in FACS buffer (PBS, 0.2% BSA and 1% sodium azide) and analyzed using a FACS flow cytometer (BD Biosciences, San Jose, CA).

### Apoptosis

Cell apoptosis was determined by an BD Biosciences Annexin V Apoptosis detection kit. Briefly, cells were collected and resuspended in 0.5 mL of binding buffer and incubated with annexin V-FITC and propidium iodide for 10 min in the dark at room temperature and analyzed using a FACS flow cytometer (BD Biosciences, San Jose, CA).

### Co-IP of proteins and immunoblot analysis

Cells were lysed with lysis buffer (20 mM Tris-HCl, 150 mM NaCl, 0.5% Nonidet P-40, 2 mM EDTA, 0.5 mM DTT, 1 mM NaF, 1 mM PMSF and 1% Protease Inhibitor Cocktail from Sigma, pH 7.5) for 30 min on ice. The lysates were cleared by centrifugation (16,000g) for 10 min at 4°C and then immunoprecipitated with anti-αII Sp antibody or IgG followed by protein G-Agarose beads. After the elution from the beads, levels of αII Sp, FANCA and XPF were analyzed by western blot.

### Metaphase spreads and chromosome analysis

Cells transfected with scrambled ncRNA, miR-128-3p mimic, miR-128-3p inhibitor or *SPTAN1* siRNA were incubated for 24 h, and subsequently treated with MMC and incubated for an additional 24 h. Colcemid (0.1 μg/ml) (Sigma-Aldrich, USA) was added 22 h after MMC treatment and incubation continued for 2 h. The cells were harvested, swollen in hypotonic solution, fixed and slides stained with 4′-6′ diamidino-2-phenylindole (DAPI). At least 100 metaphases from each group were scored for chromosomal aberrations. All fluorescence images were captured on a Nikon confocal microscope (Nikon, Tokyo, Japan) using a ×100 oil immersion lens and analyzed using Nis-element advanced research software (Nikon, Tokyo, Japan).

### Immunofluorescence

A549 cells were transfected with scrambled ncRNA, miR-128-3p mimic, miR-128-3p inhibitor or *SPTAN1* siRNA, and subsequently treated (24 h post transfection) with MMC. Then cells were applied to poly-*L*-lysine coated chamber slides and allowed to attach for 40 minutes at 37°C. They were then fixed with 4% paraformaldehyde for 20 minutes, washed with PBS and permeabilized with 0.2% Triton-X100 in PBS for 10 minutes at room temperature. The cells were then blocked in 10% FBS for 1 hours. The primary antibody of αII Sp, XPF or FANCA (Santa Cruz Biotechnology) was then added and allowed to bind for 12 hours at 4°C. After 5-minute washes with PBS, the appropriate secondary antibody was added: Alexafluor 488 mouse anti-mouse IgG conjugate (Invitrogen, Carlsbad, CA, USA), Alexafluor 594 goat anti-rabbit IgG conjugate (Invitrogen, Carlsbad, CA, USA) or Alexafluor 594 donkey anti-goat IgG conjugate (Invitrogen, Carlsbad, CA, USA). Incubation with the secondary antibodies was carried out for 1 h at room temperature in the dark and then washed with PBS for three times. The slides were then mounted with cover slips using an aqueous anti-fade mounting agent (Molecular Probes Inc.). For those cells which were examined with a DNA counter stain, after the last antibody labelling step, the cells were treated with DAPI. All fluorescence images were captured on a Nikon confocal microscope (Nikon, Tokyo, Japan) as z-stacks using a ×100 oil immersion lens and analyzed using Nis-element advanced research software (Nikon, Tokyo, Japan). Stacks were separated by 0.2–0.5 mm. All immunofluorescence staining experiments were repeated in triplicate, including negative controls to determine the background staining.

### Statistical analysis

All experiments were repeated three to five times. Data shown are presented as mean ± SE. Statistical significance was considered at *p* < 0.05 using the Student's *t*-test.

## SUPPLEMENTARY MATERIALS FIGURE


