# Supplementary material for: MicroRNA-128-3p regulates mitomycin C-induced DNA damage response in lung cancer cells through repressing SPTAN1
Source: Oncotarget. 2016 Sep 28;8(35):58098–107. doi: 10.18632/oncotarget.12300 (PMC5601636; doi:10.18632/oncotarget.12300)
Supplement: Supplementary file 1 [file oncotarget-08-58098-s001.pdf]

MicroRNA-128-3p regulates mitomycin C-induced DNA damage response in lung cancer cells through repressing *SPTAN1*

Supplementary Materials

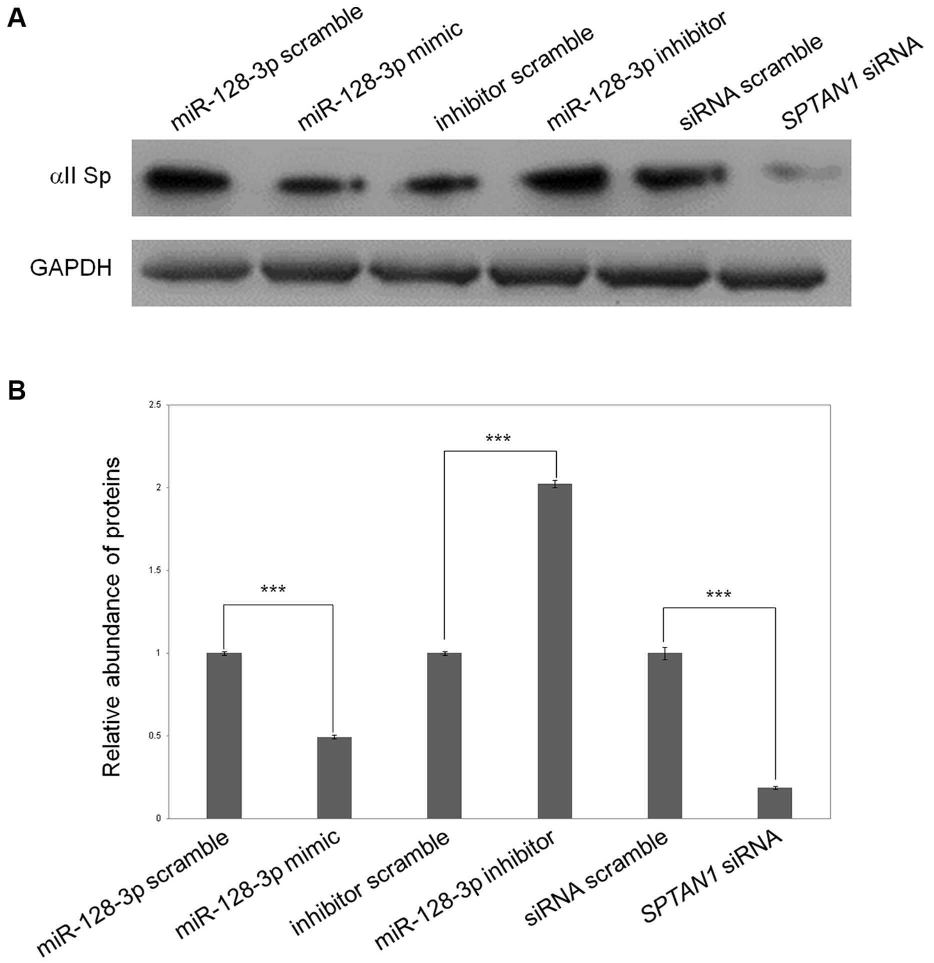

Supplementary Figure S1: MiR-128-3p regulates *SPTAN1* in H1975 cells. Western blot analysis of αII Sp protein levels in H1975 cells transfected with scrambled ncRNAs, miR-128-3p mimic, miR-128-3p inhibitor or *SPTAN1* siRNA. (**B**) Relative abundance of αII Sp. The results are presented as the mean ± SE of three independent experiments. ***$p < 0.001$.
